# Integrated Behavioral and Biological Surveillance Among People Living With HIV Visiting the Antiretroviral Therapy Centers in India: Protocol for a Cross-Sectional Surveillance

**DOI:** 10.2196/58252

**Published:** 2025-05-21

**Authors:** Pradeep Kumar, Santhakumar Aridoss, Malathi Mathiyazhakan, Subasri Dhanusu, Chinmoyee Das, Shobini Rajan, Arvind Kumar, Subrata Biswas, Elangovan Arumugam

**Affiliations:** 1 Surveillance Strategic Information Management National Organisation of AIDS Control Delhi India; 2 HIV Surveillance ICMR - National Institute of Epidemiology Chennai India; 3 Department of Computing and Information Science ICMR - National Institute of Epidemiology Chennai India

**Keywords:** people living with HIV/AIDS, antiretroviral therapy, Integrated Bio-Behavioral Surveillance, India, protocol, biological surveillance, reproductive health, sexual health, communicable diseases, mental health, integrated care, stigma, discrimination

## Abstract

**Background:**

The estimated number of people living with HIV (PLHIV) in India in 2023 is 2.54 million (range 2.16-3.03 million). With the initiation of antiretroviral therapy (ART) and the “Test and Treat” policy, the life expectancy of PLHIV on ART has substantially increased, consequently leading to a higher rate of comorbidities among PLHIV. The Joint United Nations Programme on HIV/AIDS (UNAIDS) 2025 targets aim for about 90% of PLHIV to have access to integrated and comprehensive health care services, with a concerted effort to reach the End of AIDS by 2030. Hence, the National Integrated Bio-Behavioral Surveillance (IBBS) among PLHIV (IBBS-PLHIV) has been implemented for the first time in India to establish a baseline understanding of the prevalence of sexually transmitted infections (STIs), noncommunicable diseases (NCDs), and related risk behaviors among PLHIV.

**Objective:**

The primary aim of IBBS-PLHIV is to estimate the levels of HIV-related risk behaviors and the prevalence of other STIs and NCDs among PLHIV. The specific objectives are identifying the levels of HIV-related sexual and injecting risk behaviors; estimating the prevalence of STIs such as syphilis, hepatitis B virus, and hepatitis C virus; estimating the prevalence of NCDs such as diabetes and hypertension; understanding the lifestyle and behavioral risks associated with NCDs; and assessing the levels of violence, stigma, and discrimination experienced by PLHIV.

**Methods:**

IBBS-PLHIV will be a cross-sectional, biennial surveillance among PLHIV aged 15 years or older. The first round will be implemented at 120 ART centers across 28 states, accounting for approximately 95% of the total estimated PLHIV. Consenting, eligible PLHIV will be recruited through consecutive sampling. The overall sample size at each ART center is approximately 225, and the surveillance period is 3 months. Behavioral data on demographics, reproductive and sexual health, lifestyle and sexual behaviors, stigma, and discrimination will be collected. Blood samples will also be collected to test for STIs and NCDs.

**Results:**

IBBS-PLHIV was initiated on January 1, 2024, in a phased manner. Data collection was carried out over 3 months and completed by June 2024 across all 120 sites. A total of 25,257 PLHIV were recruited for the surveillance, including 11,921 males, 11,855 females, and 1481 hijra/transgender individuals. Data entry, followed by data matching and validation of all records, was completed in December 2024. The data are currently being analyzed, and the final findings are expected to be disseminated by December 2025.

**Conclusions:**

Data collected through IBBS-PLHIV will help monitor the levels of HIV-related sexual and injecting risk behaviors among PLHIV. Additionally, it will provide estimates of the prevalence of NCD comorbidities and STI coinfections such as diabetes, hypertension, syphilis, and viral hepatitis. These findings will serve as a baseline and are expected to offer valuable insights for facilitating comprehensive HIV care and management through the effective integration of HIV and broader health service delivery.

**International Registered Report Identifier (IRRID):**

DERR1-10.2196/58252

## Introduction

### Background

The HIV/AIDS epidemic is highly concentrated and confined to specific population groups and geographical locations. In India, the predominant mode of HIV transmission is through the heterosexual route (75%), followed by injecting drug use (12%), and the homosexual or bisexual route among men who have sex with men (5%) [1]. In India, as per the 2023 report, the estimated adult prevalence of HIV is 0.20% (95% CI 0.17%-0.25%), and the estimated number of people living with HIV (PLHIV) is 2.54 million (95% CI 2.17-3.04 million) [2]. Of these, about 2.48 million (97.5%) total infections were among adults aged 15 years and above. The estimated number of PLHIV per million population was 1841 [2].

The Joint United Nations Programme on HIV/AIDS (UNAIDS) is leading the global agenda to eliminate HIV/AIDS as a public health threat by 2030. The UNAIDS 2025 strategy emphasizes 3 targets for HIV services, focusing on the Sustainable Development Goals related to the HIV response [3]. The primary target is to achieve the 95-95-95 testing and treatment goals among all subpopulations and age groups of PLHIV by 2025. Accordingly, 95% of PLHIV should know their HIV status, 95% of those who know their status should be on antiretroviral therapy (ART), and 95% of those on ART should have suppressed viral loads by 2025. The strategy also emphasizes eliminating social and legal barriers to HIV services and integrating these services with other health services for PLHIV. By achieving the 2025 targets, it is expected that at least 90% of PLHIV and individuals at higher risk of HIV infection will be linked to integrated services that support their overall health and well-being [3,4].

As part of the National AIDS Control Programme (NACP) of the National AIDS Control Organisation (NACO), the free ART initiative was launched in 2004 in Delhi and 6 other high-prevalence states. Since then, ART services have been scaled up and decentralized to ensure access for all PLHIV. With the adoption of the Treat All policy in 2017, all PLHIV are initiated on ART regardless of CD4 count, clinical stage, or age. About 81 % of PLHIV in India know their HIV status, of whom 88% are on ART; among those, >= 95% have suppressed viral loads—translating to approximately 69% of all PLHIV having suppressed viral loads [1].

Noncommunicable diseases (NCDs) are emerging as a significant cause of morbidity and mortality among PLHIV. The increasing incidence of NCDs can be attributed to various factors, including chronic immune activation, medication side effects, coinfections, and the aging of the PLHIV population. Promoting awareness of NCDs through health education and early detection and diagnosis, followed by treatment and regular follow-up, is recommended to prevent and manage NCDs in this group. With the growing availability and accessibility of ART centers, many PLHIV are benefiting from integrated HIV care services [5].

HIV management and care services are provided to approximately 1.7 million PLHIV through 775 ART centers, 1307 link ART centers, and 320 partner-supported care and support centers [1]. However, stigma, lack of awareness, and missing linkages between testing and treatment services remain significant challenges to advancing the End of AIDS agenda. Additionally, the lack of data on key indicators, such as viral suppression rates among high-risk groups, makes it difficult to design effective health programs that address the specific needs of populations affected by the HIV epidemic in the country [6]. To overcome these challenges, improved surveillance of comorbidities among PLHIV within an integrated HIV care setting and the strengthening of existing data systems are highly recommended [7,8]. HIV Sentinel Surveillance (HSS), one of the earliest interventions under the NACP, has played a crucial role in characterizing the HIV epidemic and reinforcing HIV prevention and control efforts in India. HSS is an ongoing, cross-sectional surveillance activity conducted biennially to monitor the levels and trends of HIV prevalence across the country. It is implemented among 8 population groups: pregnant women, prisoners, migrants, truckers, female sex workers, men who have sex with men, hijra/transgender (H/TG) individuals, and injecting drug users. In alignment with the UNAIDS 2025 targets, the National AIDS and STD Control Programme has developed the Integrated and Enhanced Surveillance and Epidemiology (IESE) framework to study the epidemiology of HIV and other related sexually transmitted infections (STIs) among all high-risk populations. Under the IESE framework, a periodic cross-sectional Integrated Bio-Behavioral Surveillance among PLHIV (IBBS-PLHIV) was implemented in 2024 for the first time in India. This surveillance aims to collect epidemiological data using a scientifically rigorous methodology, guided by standard ethical considerations. This paper describes the objectives, detailed methodology, and data management plan of the IBBS-PLHIV surveillance, along with its expected outcomes.

### Aims and Objectives of IBBS-PLHIV

The overall aim of the IBBS among PLHIV is to monitor the levels, trends, and determinants of HIV-related behaviors, service uptake, and comorbidities among PLHIV, to support improved planning of integrated care and treatment services.

Specific objectives are as follows:

- To generate evidence on HIV-related risk behaviors, such as condom use, number of partners among PLHIV, injecting drug use, and injecting practices.
- To estimate the prevalence of related STIs, namely, syphilis, hepatitis B, and hepatitis C, among PLHIV.
- To understand lifestyle behaviors and estimate the prevalence of associated NCDs, including diabetes, hypertension, heart disease, chronic respiratory illness, chronic liver disease, chronic kidney disease, and cancer.
- To assess the levels of violence, stigma, and discrimination experienced by PLHIV.

## Methods

### Surveillance Design and Setting

The IBBS-PLHIV is proposed as a biennial, cross-sectional surveillance survey among PLHIV aged 15 years and older. The survey is planned to be conducted over 3 months, from January to March, every 2 years until 2030.

For the first round, the surveillance will be conducted at 120 designated ART centers across 28 states, which account for approximately 95% of the total PLHIV burden in the country. The recruitment process will begin on January 1, 2024, and is expected to be completed by March 31, 2024, or earlier if the desired sample size is achieved.

### Sample Size and Sampling Approaches

The sample size for each domain is calculated to measure consistent condom usage among adult PLHIV by gender. A domain refers to a geographical unit for which representative estimates will be generated.

In an experimental study conducted among HIV-positive women in 2 tertiary care hospitals in Mumbai [9], consistent condom use among spouses of HIV-positive women was 77%, which increased to approximately 96% in the intervention arm over 1 year. Based on this context, the sample size required to measure the trend has been calculated according to the considerations outlined below:



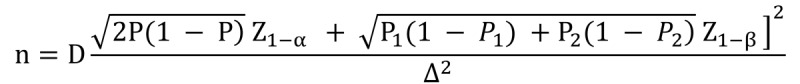



where *D* is the design effect; *P*_1_ is the estimated proportion at the time of first surveillance (expected baseline value); *P*_2_ is the estimated proportion at some future date, so that (*P*_2_~*P*_1_) is the magnitude of change needed to be detected (expected value after 2 years); *P*=[(*P*_1_+*P*_2_)/2]; *Z*_1–_*_α_* is the *z*-score corresponding to the desired level of significance (1-sided); *Z*_1–_*_β_* is the *z*-score corresponding to the desired level of power; and Δ^2^ = (*P*_2_~*P*_1_)^2^.

Consistent condom use with a spouse is used as a key indicator to calculate the sample size for the surveillance population. The expected baseline value (*P*_1_) and the expected magnitude of change (*P*_2_) are 77% and 90%, respectively, based on the Mumbai Study [9]. *P*_2_ is kept lower than the 96% observed in the Mumbai study, as that was conducted in a research setting. *Z*_1–_*_α_* is 1.64 at a 95% CI, and *Z*_1–_*_β_* is 0.84 at 80% power, with a design effect of 2.

Based on the above equation and the parameters, the sample size was calculated to be 198, rounded up to 200. The IBBS-PLHIV surveillance aims to measure changes in the study variables separately for males and females. Therefore, the sample size is set at 200 males and 200 females for each surveillance domain. The surveillance will also include H/TG individuals as one of the surveillance groups. The number of H/TG individuals accessing ART centers is expected to be low due to migration and loss to follow-up. Therefore, a “take-all” approach will be implemented across selected ART centers for H/TG participants to ensure their participation.

The surveillance sites and subsites in the selected state or union territory will be chosen to spatially represent the established administrative or sociocultural regions, based on the laboratory facilities available at the selected sites. In the first round, 120 ART centers across 7 geographical regions (North, Central, West, East, Northeast, Southeast, and South) of India have been selected. During the IBBS-PLHIV, 1 premier public health research institute will be designated as the regional institute (RI) to provide technical assistance and supportive supervision at the sites within the states allocated to them (Table 1). All districts in each state will be grouped into 2-4 geographical domains, based on their geographical and sociocultural characteristics, to ensure that ART centers from each domain are included. Considering the wider spread and feasibility of implementation, 2 ART centers will be selected from each domain. For smaller states, such as Goa, the entire state will be considered a single geographical domain. As the calculated sample size is 200 for each domain and 2 ART centers are selected from each domain, the target sample size for each ART center will be 100 males, 100 females, and approximately 25 H/TG individuals. According to the 2023 HIV estimation, approximately 43% of PLHIV in India are females. Therefore, an equal gender distribution was set, also considering the feasibility of a multicentric surveillance that is planned to take place at regular intervals. Similarly, for the H/TG population, an estimate of H/TG individuals registered at each ART center was determined before the survey, which ranged up to 25. To include the largest possible H/TG population, an upper limit of 25 was set for each ART center, and a take-all approach will be followed. The findings for the H/TG population will be reported only as national aggregates. However, any findings reported will be based on weighted analysis to account for potential differences. Therefore, the overall sample size for PLHIV surveillance will be approximately 27,000 nationally, with around 4500 (2000 males, 2000 females, and 500 H/TG individuals) for each region in 1 round. This sample size will be sufficient to provide robust prevalence estimates of coinfections at the regional level, with an anticipated prevalence of 1% and an absolute precision of 0.5% for males and females separately. For the H/TG population, the sample size will be adequate at the national level to provide estimates of coinfections, provided that approximately 25 H/TG individuals are recruited at each ART center.

**Table 1:** Representation of antiretroviral therapy sites to be included in the surveillance.

Region, regional institute, and states/union territory				Antiretroviral therapy centers (N=120)
**North**				
	**PGIMER^a^, Chandigarh (4 states)**			
		Chandigarh	1	
		Haryana	2	
		Himachal Pradesh	2	
		Punjab	4	
**Central**				
	**AIIMS^b^, New Delhi (4 states)**			
		Delhi	4	
		Jharkhand	3	
		Uttar Pradesh	8	
		Uttarakhand	3	
**West**				
	**ICMR^c^—NITVAR Pune (6 states)**			
		Goa	2	
		Gujarat	4	
		Madhya Pradesh	4	
		Maharashtra	8	
		Mumbai	2	
		Rajasthan	6	
**Northeast**				
	**RIMS^d^, Imphal (3 states)**			
		Manipur	3	
		Mizoram	4	
		Tripura	3	
**East**				
	**ICMR—NIRBI^e^ Kolkata (5 states)**			
		Assam	3	
		Meghalaya	3	
		Nagaland	3	
		Chhattisgarh	8	
		West Bengal	6	
**Southeast**				
	**AIIMS, Bhubaneswar** **(2 states)**			
		Odisha	3	
		Andhra Pradesh	6	
**South**				
	**ICMR—NIE^f^ Chennai (4 states)**			
		Kerala	3	
		Karnataka	8	
		Tamil Nadu	8	
		Telangana	6	

^a^PGIMER: Postgraduate Institute of Medical Education and Research.

^b^AIIMS: All India Institute Of Medical Sciences.

^c^ICMR: Indian Council of Medical Research.

^d^RIMS: Regional Institute of Medical Sciences.

^e^NIRBI: National Institute for Research in Bacterial Infections.

^f^NIE: National Institute of Epidemiology.

A consecutive sampling method will be used for all eligible and consenting PLHIV at selected ART centers to minimize selection bias. ART centers follow a standardized patient flow, and every PLHIV visiting the ART center is registered in the daily register. The sampling process begins at this point of daily registration, from where PLHIV will be directed to the medical officer in the order they visit the ART center on a given day. The medical officer will verify eligibility and direct eligible PLHIV to the counselor for recruitment into IBBS-PLHIV.

The sample size at each ART center is approximately 225, with a surveillance period of 3 months. Therefore, a maximum of 5 respondents will be recruited daily to ensure the quality of the collected data and samples. However, because a take-all approach is followed, if 1 or more H/TG PLHIV is present after the fifth recruitment, they will be included in the surveillance to ensure their participation. Samples will be collected until the end of the surveillance period or until the desired sample size is achieved. The key features of IBBS among PLHIV, based on the population groups, are shown in Table 2.

**Table 2:** Key features of the Integrated Bio-Behavioral Surveillance among people living with HIV by population groups.

Key population group	Description
Setting	A facility based at designated antiretroviral therapy centers
Sample size (per antiretroviral therapy center): female/male/hijra/transgender	100/100/25
Sampling methodology	Consecutive sampling
Behavioral data domain	Demographic characteristics, family planning practices (for males and females), child birth history (only for females), lifestyle behaviors, sexual health, stigma, discrimination, and violence
Clinical data	Height, weight, and systolic and diastolic blood pressures
Biological specimen	Venous blood
Biomarkers	Hepatitis B virus, hepatitis C virus, and syphilis

### Characteristics of Participants

#### Inclusion Criteria

Males, females, or H/TG individuals with HIV/AIDS, aged 15 years or older, who are registered at the designated ART center, and visit the ART center during the surveillance period are eligible for recruitment in the surveillance. Only eligible PLHIV who provide written consent to participate will be included in the surveillance.

#### Exclusion Criteria

PLHIV under 15 years of age, those not registered at the designated ART center, or those who have already participated in the current round of surveillance will be excluded from the surveillance.

### Data Collection

A written informed consent will be obtained from all eligible PLHIV who are willing to participate in the surveillance. Bio-behavioral data will be collected from all consenting respondents using a paper-based tool in the form of a structured questionnaire (Multimedia Appendices 1-3). Data on demographic characteristics, reproductive and sexual health behaviors, lifestyle behaviors, and experiences of stigma, discrimination, and violence will be collected, as outlined in Table 3. Additionally, clinical data such as height, weight, and systolic and diastolic blood pressures will be measured using standard instruments.

**Table 3:** An overview of the surveillance variables to be collected.

Surveillance variables	Population group		
	Female	Male	Hijra/transgender
Background characteristics: age, literacy, marital status, and occupation	✓	✓	✓
Reproductive health: family planning practices	✓	✓	✗
Fertility: birth history, current pregnancy, and breastfeeding practices	✓	✗	✗
Sexual history: age at first sexual intercourse, recent sexual activity, number and type of sexual partners, and condom use	✓	✓	✓
Lifestyle behaviors, prevalence of comorbidities, and treatment	✓	✓	✓
Stigma and discrimination	✓	✓	✓

### Sample Collection

Venous blood samples will be collected to test for CD4 count, viral load, random blood sugar, and biomarkers for other STIs, such as syphilis, hepatitis B virus, and hepatitis C virus. The CD4 count, viral load, and random blood sugar will be tested according to the routine guidelines followed at the ART center (Multimedia Appendix 4). To test for other STIs, blood samples will be processed according to the laboratory protocol to obtain serum. The serum will be stored at 2-8°C until transportation and sent weekly to designated laboratories for testing. The results of all reactive tests will be returned to the respondents through the ART centers for follow-up and treatment.

### Testing Strategy

A linked anonymous testing strategy will be adopted for testing the blood specimens collected during IBBS-PLHIV. A 2-test protocol will be used for testing syphilis: rapid plasma reagin and *Treponema* pallidum hemagglutination assay. A 1-test protocol will be used for testing hepatitis B virus and hepatitis C virus, using rapid antigen testing kits (Multimedia Appendix 4). According to the Guidelines for Surveillance of Sexually Transmitted Diseases, diagnostic tests are most effective for assessing disease prevalence when they specifically detect active infections. Treponemal tests alone, which typically remain reactive for life, are insufficient for distinguishing between adequately treated and active syphilis infections. Therefore, a 2-test protocol is recommended for syphilis surveillance, incorporating a nontreponemal test with a titer cut-off (eg, 1:4 or 1:8) to monitor active infection trends [10].

By contrast, the surveillance of hepatitis B and C aims to estimate the disease burden of chronic infections. For this purpose, viral hepatitis screening is conducted using a qualitative 1-test protocol, as endorsed by the National Viral Hepatitis Program [11].

### Implementation Plan and Structure

NACO will implement the surveillance activity at designated ART centers in selected states across the country. An implementation structure similar to the HSS among ANC attendees will be adopted. The State AIDS Control Society (SACS) and the respective District AIDS Prevention and Control Units/District Integrated Strategy for HIV/AIDS Units will coordinate and implement the activity at the state level. Three site personnel—a medical officer, a counselor or nurse, and a laboratory technician—will be involved in the surveillance activity at each site.

The ART medical officer will be responsible for the PLHIV surveillance at the ART center. The medical officer will verify the eligibility of the PLHIV based on the inclusion criteria and direct eligible individuals to the counselor. The counselor will confirm the eligibility of the PLHIV and, if found eligible, will administer the participant information sheet (PIS), informed consent form (ICF), and assent forms, as applicable. The counselor will collect behavioral data from all consented, eligible PLHIV using the data forms and maintain the forms until they are dispatched to the respective RIs. Additionally, the counselor will be responsible for filling out and maintaining the HSS register in a secure and confidential manner to ensure linked anonymous testing. The counselor will also oversee the dispatch of the data forms and registers according to the guidelines. The laboratory technicians will be responsible for measuring clinical data, such as height, weight, and blood pressure. The technician is primarily responsible for collecting and processing blood samples according to the protocol. The lab technician is also in charge of storing and transporting the samples to the linked surveillance laboratory. A completed data form, labeled with the state code, site code, sample number, and date of collection, along with 2 data form transportation sheets, will be sent to the respective RI every week. Similarly, the processed samples, properly labeled and packed, will be sent to the testing laboratories each week.

Overall, technical and monitoring support will be provided by national and regional institutes, SACS, and state surveillance teams. The activity will be implemented under the guidance of NACO’s Technical Working Group for Surveillance & Epidemiology, in collaboration with bilateral and multilateral partners, as well as other relevant stakeholders. The respective SACS will be responsible for procuring and disseminating the surveillance materials to the ART centers. Procurement will be carried out according to the technical specifications set by the nodal agency to ensure quality and uniformity. The materials will be procured and disseminated before the initiation of the survey. Similarly, training for site personnel will be conducted by the SACS 1 week before the survey’s start. An extension may be granted on a case-by-case basis to any sites that are unable to achieve the sample size within the surveillance period. In the event of termination of the surveillance at a particular site or domain due to unforeseen circumstances, data from that domain will not be included in the analysis if the sample collected up to the point of termination is insufficient. However, tests will be performed on the collected samples, and appropriate services will be linked for any positive results.

### Ethical Considerations

#### Voluntary Participation

Participation in the surveillance is voluntary and free from coercion. Eligible respondents who refuse to participate will not be forced, and routine services will be provided. However, the reasons for refusal will be documented in the IBBS-PLHIV register.

#### Confidentiality

Any personal identifiers, such as name, contact number, address, and clinical diagnosis, will not be accessible to anyone except the site-level personnel involved in the surveillance. Confidentiality will be ensured by assigning a unique identification number to each participant. Any diagnostic results generated will be delinked from personal identifiers. A separate link register, containing only the respondent’s ART number and surveillance sample number (with no other personal identifiers), will be maintained to facilitate follow-up service delivery. The delinked data will be accessible only to designated members of the surveillance team at the state, regional, and national levels. The anonymous data will be used for analysis, reporting, publication, and policy making. Only summarized, anonymized data will be used for publication. Additionally, any reporting required by government agencies, as per the law, will be conducted periodically. To ensure data privacy and confidentiality at each level, access to the data management portal is user specific. Each individual involved in the surveillance will be provided with unique log-in credentials, and their access will be restricted based on role-specific permissions. The database will be accessible only to authorized personnel at the state, regional, and national levels, and national guidelines for data management will be strictly followed.

#### Participant Information Sheet and Informed Consent

All eligible PLHIV will be provided with a PIS and written ICFs before data collection. The PIS will cover the objectives of this PLHIV surveillance, expectations from the respondents, information on biological test results, confidentiality, and voluntary participation. Written ICF will be obtained from sampled and eligible participants who are willing to participate in the surveillance. If the eligible participant is literate, the PIS and ICF will be provided for them to read. If the participant is illiterate, the PIS and ICF will be read aloud in the presence of a literate witness. The PIS and ICF will be translated into local languages. Any queries will be addressed promptly and adequately. Respondents will be shown all consumables and items used for biological sample collection and assured of confidentiality. For respondents aged 15-17 years, assent will be obtained along with the consent of a parent, guardian, or health care provider not associated with the survey.

#### Diagnosis and Treatment

All respondents who test reactive for any of the biomarkers will be contacted and informed of their diagnosis. They will be referred for appropriate treatment as per program guidelines.

The ethical considerations and informed consent process for the IBBS-PLHIV were reviewed and approved by the Institutional Ethics Committee of ICMR—National Institute of Epidemiology. The approval number is NIE/IHEC/202207-03, dated August 23, 2023.

### Data Management

Data will be collected using prestructured, paper-based forms. These forms will be bilingual, with questions in both English and the regional language. The forms will be translated and pilot-tested before implementation. All collected data forms, samples, and related documents will be handled exclusively by designated surveillance team members at all levels.

Surveillance and data management will be conducted through a customized web-based portal. The portal is designed to facilitate the entry and updating of all surveillance activities, including site preparedness, daily data/sample collection status, transportation sheets, acknowledgment of data/sample receipts, sample checklists, laboratory results, supervisory visit forms, and surveillance data. Role-based access control is implemented to ensure data confidentiality and security. As a result, all entries can be made and managed only by designated users in a secure manner.

The designated RIs will manage data at the regional level. Upon receipt of the data forms, the data manager will ensure and acknowledge their safe arrival. Data entry operators will enter the data into the web-based portal. All data will be synced to a central server, allowing access from various sites/centers. Double data entry will be performed for all forms to minimize errors. Any personal identifiers (if present) will be delinked from the captured data to ensure confidentiality.

All personnel involved in data management and data entry will be appropriately trained to minimize errors. The data manager will also be responsible for cleaning and validating the entered data for further analysis. The data will be periodically monitored by the data manager to ensure consistency. Laboratory results will be directly entered into the portal by the linked surveillance laboratory. These results will be matched with the cleaned behavioral data using the IBBS number (12 digits) as a unique identifier. To ensure data integrity, data matching will be performed exclusively by the designated in-charge at the RI.

### Data Analysis Plan

The matched data will be precoded for analysis, and missing data will be coded accordingly. The data will be analyzed to estimate the levels of sexual risk behaviors, the prevalence of STIs/NCDs, and associated behaviors among PLHIV. Results will be presented using descriptive statistics. Where required, means/medians, differences in means/medians, proportions, and their corresponding 95% CIs will be presented. Data from the first round of the PLHIV surveillance will be used to establish a baseline for the current HIV interventions, with subsequent rounds compared to assess the impact of integrated services. The sexual behavioral risks associated with HIV-related STIs and the lifestyle behavioral risks associated with NCDs among PLHIV will be analyzed using regression methods. All statistical analyses will be conducted using standard statistical software, and values with *P*<.05 will be considered significant. A weighted analysis will be performed to account for significant differences in PLHIV burden and sample size. A more detailed analysis plan for key indicators is provided in Multimedia Appendix 5.

### Quality Assurance at the Surveillance Sites and Testing Laboratory

#### Overview

Training for surveillance activities, along with monitoring by the state, regional, and national teams, and interlaboratory comparison, will be conducted as part of quality assurance to minimize errors.

#### Training of the Site Personnel

As part of the IBBS-PLHIV, a 2-day in-facility training will preferably be conducted for the ART surveillance team at the site level to build their capacity on survey procedures. The training modules will cover data form collection and transportation, sample collection, processing, packaging, and transportation. Practical sessions on “Know Your Sentinel Site” and IBBS methodology will be included to ensure the proper implementation of the IBBS workflow.

#### Monitoring and Supervision

Trained surveillance team members at the state, regional, and national levels will monitor the survey. A supportive supervisory visit to all sites will be conducted within the first week of the surveillance to facilitate the identification and correction of any errors in advance. Adherence to the IBBS methodology, as well as the quality of data and sample collection, sample processing, and storage, will be ensured during supervisory visits. The first few forms will be thoroughly checked for data quality control to prevent further mistakes. In addition to on-site visits, the RIs and designated state surveillance team members will virtually monitor the sites for data and sample collection quality assurance. The data and sample collection status of each site will be monitored daily via virtual platforms. Similarly, periodic reviews of data forms, the PLHIV surveillance register, and the link register will be conducted virtually. The RI will provide refresher training in case any discrepancies are identified.

#### Quality Assurance at Testing and Reference Laboratories

The samples will be transported to designated testing laboratories weekly. Upon receipt of the samples, the laboratory in charge will perform a quality check on all received samples using the sample quality checklist. The status of accepted and rejected/invalid samples will be promptly communicated to the RI and the respective sites.

Interlaboratory comparisons will be conducted to ensure testing quality. All serum specimens that are positive for 1 or more biomarkers, along with 5% of negative specimens (those not positive for any of the biomarkers), will be sent to a national reference laboratory for quality assurance testing every fortnight. The negative specimens will be randomly selected.

### Data Dissemination

The observations and surveillance results will be presented as top-line findings, technical briefs, national and state reports, and scientific publications. Publications based on these findings may be presented at scientific conferences and in peer-reviewed journals, in consultation with the Ministry of Health and Family Welfare, Government of India and with input from the technical resource group and technical working group of NACO. The findings from the surveillance survey will be presented to policy makers to inform program design, set benchmarks, and assess progress over time.

## Results

The IBBS-PLHIV was initiated on January 1, 2024, in a phased manner. Data collection was conducted over 3 months and completed by June 2024 at all 120 sites. A total of 25,257 PLHIV were recruited for the surveillance, including 11,921 males, 11,855 females, and 1481 H/TG individuals. Data entry, followed by data matching and validation of all records, was completed in December 2024. The data are currently being analyzed, and the final findings are expected to be disseminated by December 2025. The state-wise gender distribution of PLHIV respondents is shown in Table 4.

**Table 4:** State-wise gender distribution of people living with HIV respondents in the Integrated Bio-Behavioral Surveillance-People Living With HIV Program, 2024.

State	Female (n=11,855), n	Male (n=11,921), n	Hijra/transgender (n=1481), n	Overall, n
Andhra Pradesh	599	601	150	1350
Assam	301	303	18	622
Chandigarh	102	98	25	225
Chhattisgarh	807	794	81	1682
Delhi	410	430	104	944
Goa	200	200	3	403
Gujarat	401	400	64	865
Haryana	199	201	27	427
Himachal Pradesh	200	201	5	406
Jharkhand	301	298	29	628
Karnataka	797	805	88	1690
Kerala	301	299	22	622
Madhya Pradesh	398	416	7	821
Maharashtra	1009	1007	172	2188
Manipur	303	301	50	654
Meghalaya	202	203	0	405
Mizoram	468	459	0	927
Nagaland	298	302	0	600
Odisha	294	295	72	661
Punjab	404	397	77	878
Rajasthan	598	602	34	1234
Tamil Nadu	805	810	140	1755
Telangana	601	599	87	1287
Tripura	254	300	1	555
Uttar Pradesh	700	703	109	1512
Uttarakhand	300	300	16	616
West Bengal	603	597	100	1300

## Discussion

### Expected Findings

The IBBS-PLHIV will be a biennial cross-sectional survey aimed at monitoring the levels, trends, and determinants of HIV-related behaviors and comorbidities among PLHIV. Anticipated outcomes of IBBS-PLHIV include identifying HIV-related sexual behavioral risks, such as inconsistent condom use and partner types, as well as estimating the prevalence of HIV-related STIs, including syphilis, hepatitis B, and hepatitis C. The surveillance also aims to assess lifestyle behavioral risks such as smoking, alcohol consumption, and injecting drug use, as well as the self-reported prevalence of NCDs such as diabetes, hypertension, heart disease, chronic respiratory illness, chronic liver disease, chronic kidney disease, and cancer. Additionally, participants will be screened for diabetes through random blood sugar measurements and for hypertension through the measurement of systolic and diastolic blood pressure. Furthermore, the surveillance aims to assess the reproductive and sexual health needs of PLHIV, as well as the levels of violence, stigma, and discrimination they experience. To understand how the findings from the surveillance will improve HIV management among PLHIV, it is essential to examine the existing health care and HIV management cascade in India.

The health care system in India is diverse, with both public and private health care service providers. Rural areas are primarily served by the 3-tiered public health care system, which includes primary, secondary, and tertiary levels of care, while private providers predominantly cater to urban areas. The PHCs and subcenters are primary health care units that primarily focus on integrated preventive and curative care for the rural population. Secondary-level units, such as subdistrict hospitals and community health centers, serve as referral units. Tertiary-level care is provided by district hospitals and medical colleges. Both public and private providers contribute to secondary and tertiary health care services [12]. Similarly, a multitier model of HIV care is provided by the ART centers. Primary care is offered by ART centers and facility integrated ART centers located at government medical colleges, district hospitals, selected subdistrict hospitals, and private institutes. Secondary care is provided by ART Plus centers, while specialized care is offered at Centers of Excellence in selected medical colleges. Additionally, decentralized care is provided through link ART centers at subdistrict hospitals and community health centers, care and support centers, opioid substitution therapy centers, prisons, and targeted intervention sites [13]. In India, 775 ART centers, 1307 link ART centers, and 320 partner-supported care and support centers provide HIV management and care services to nearly 1.7 million PLHIV [1]. Additionally, a comprehensive care support and treatment service package includes free diagnostic and monitoring services, with an emphasis on long-term engagement in care, proactive management of opportunistic infections, and linkages to support services and social protection schemes. In summary, it ensures accessible and lifelong ART while addressing various aspects of care and social protection [5].

ART centers adhere to the Test and Treat policy, ensuring universal and unrestricted access to ART for PLHIV, regardless of their CD4 count or demographic factors. A comprehensive HIV cascade, including HIV testing, treatment, and care, has been globally adopted to achieve targeted viral suppression among PLHIV. Recent research highlights the benefits of early initiation of ART in improving the health of PLHIV and preventing HIV transmission to partners, thereby reducing HIV incidence. As a result, most HIV prevention programs have focused on evaluating and enhancing PLHIV engagement in the HIV cascade of testing, treatment, and care. Yet, only about 69% of the total PLHIV in India have their viral load suppressed, indicating substantial gaps despite the progressive estimates at the national level. Recent studies report an increase in high-risk sexual behavior among the general population, particularly among youth, urban populations, and females, who face disproportionately increased vulnerabilities to HIV infection in India [14-16]. While NACO has intensified treatment services, prevention remains the cornerstone of India’s HIV response, emphasizing the need for monitoring risk behaviors among PLHIV to achieve zero transmission. Although some researchers have reported evidence of improved clinical and behavioral indicators based on retrospective analysis of PLHIV data, second-generation surveillance is globally recommended to identify the dynamics of HIV risk behaviors to improve prevention and treatment outcomes [17-19].

By contrast, despite declining HIV infections, the widespread use of ART has transformed HIV into a chronic condition, reducing AIDS-related deaths but increasing the prevalence of other comorbid conditions among PLHIV. India faces a dual burden of HIV and NCDs, driven by aging, the long-term effects of HIV, and ART [20]. Several studies in India have reported metabolic changes; insulin resistance; and increased risks of myocardial infarction, stroke, liver disease, kidney disease, and cancers, including non–AIDS-defining cancers, among PLHIV [21-27]. Similarly, a higher prevalence of STI coinfections is also being documented in significant proportions [28,29]. Further, evidence suggests that PLHIV frequently encounter stigma and discrimination due to their HIV status, both within their communities and health care settings, leading to the underutilization of HIV-related services [6,30,31]. In contrast to the decentralized, vertical HIV service delivery model, NCD and STI services are predominantly centralized through hospitals. While relevant national programs exist for each of the comorbidities, gaps in service delivery, human resources, and monitoring persist. India’s HIV program focuses on continuous care and ART adherence; however, limitations remain in the structured integration with NCD/STI management [20]. Although a referral mechanism is in place at all ICTC and ART centers, where PLHIV are informed and referred to the nearest NCD/STI facility for linkage and treatment, resource limitations hinder follow-up and the provision of integrated care at ART centers [21]. Recent studies have underscored the need for enhanced data collection and surveillance of NCDs, STIs, associated behaviors, and the stigma and discrimination experienced by PLHIV to identify the limitations of existing programs [7,20]. Strengthening routine data collection and patient monitoring systems is essential for comprehensive HIV management, thereby addressing global health inequities.

In light of this, a robust IBBS among PLHIV has been pioneered in India under the IESE framework to oversee the management and advancement of public health response initiatives under NACP. Given the periodic nature of the surveillance, the data can be used to monitor the levels and trends of health conditions among PLHIV and evaluate the progress of program interventions. Data generated from the surveillance will ultimately lead to informed decisions for providing holistic HIV management services at ART centers, ensuring regular screenings for other infections and chronic conditions, facilitating more effective linkages and treatment, and addressing stigma.

### Limitations

The surveillance will be implemented in 28 states across India, covering 95% of the country’s HIV burden. It will be conducted at ART centers in government facilities and will include both PLHIV already registered and new registrants visiting the centers during the surveillance period. As mentioned, of the estimated 2.5 million adult PLHIV, only 1.7 million are on ART. As the surveillance is facility based, PLHIV who have never registered at ART centers will not be included in the sampling frame. This limits data collection from hidden populations of PLHIV who are unlinked to HIV/ART services, necessitating appropriate caution in interpreting the results. Likewise, the surveillance will not capture data from PLHIV accessing ART services through private facilities. The sample size is calculated to generate national- and state-level estimates; therefore, appropriate caution must be exercised when interpreting results at the district level.

## Appendix

Multimedia Appendix 1 IBBS-PLHIV—female data form. IBBS: Integrated Bio-Behavioral Surveillance; PLHIV: people living with HIV.

Multimedia Appendix 2 IBBS-PLHIV—male data form. IBBS: Integrated Bio-Behavioral Surveillance; PLHIV: people living with HIV.

Multimedia Appendix 3 IBBS-PLHIV—hijra/transgender data form. IBBS: Integrated Bio-Behavioral Surveillance; PLHIV: people living with HIV.

Multimedia Appendix 4 Summary of the biospecimens, biomarkers, and testing strategy.

Multimedia Appendix 5 Key indicators: data analysis plan.

## Abbreviations

ART: antiretroviral therapy
H/TG: hijra/transgender
HSS: HIV Sentinel Surveillance
IBBS: Integrated Bio-Behavioral Surveillance
ICF: informed consent form
IESE: Integrated and Enhanced Surveillance and Epidemiology
NACO: National AIDS Control Organisation
NACP: National AIDS Control Programme
NCD: noncommunicable disease
PIS: participant information sheet
PLHIV: people living with HIV
RI: regional institute
SACS: State AIDS Control Society
STI: sexually transmitted infection
UNAIDS: Joint United Nations Programme on HIV/AIDS

## Data Availability

All individual-level data collected during the surveillance will be maintained by the National AIDS Control Organization (NACO), in accordance with its Data Management and Data Protection Guidelines. Data may be shared with requesting individuals or organizations upon approval by the competent authority, following the proper request process outlined in the guidelines available at [32].
