# Supplementary material for: Integrated Behavioral and Biological Surveillance Among People Living With HIV Visiting the Antiretroviral Therapy Centers in India: Protocol for a Cross-Sectional Surveillance
Source: JMIR Res Protoc. 2025 May 21;14:e58252. doi: 10.2196/58252 (PMC12138296; doi:10.2196/58252)
Supplement: Multimedia Appendix 3 [file resprot_v14i1e58252_app3.pdf]

ANNEXURE 3

IBBS-PLHIV - Transgender / Hijra Data Form

INTEGRATED BIOLOGICAL AND BEHAVIOURAL SURVEILLANCE AMONG PEOPLE LIVING WITH HIV/AIDS

Operational Definition:

Any HIV-positive Transgender / Hijra aged 15 years and above registered and visiting the ART centre during the surveillance period.

IDENTIFICATION SECTION

State: ____________________District: ____________________<br>Site Name: ____	
(Site Code) ☐☐☐☐☐☐☐☐ (Subsite) ☐ (Sample No) ☐☐☐ (Date DD/MM/YY) ☐☐☐☐☐☐	

CONSENT STATUS

Administer the informed consent form to the selected respondent. Follow all the guidelines for administering informed consent. **Record** this consent status **before starting the interview.**

**CONSENT**	**Circle appropriate options**	
I consent for the survey team to interview.	1. Yes	2. No
I consent to provide the blood sample and get it tested for Syphilis, Hepatitis, Hepatitis C, CD4 count, HIV viral load, and Random blood sugar.	1. Yes	2. No
I consent to be contacted for follow-up medical services if my samples are reactive to any of the tests.	1. Yes	2. No
I consent to the publication and dissemination of anonymized and combined data.	1. Yes	2. No
I consent to store the left-over (if any) samples for public health activities.	1. Yes	2. No

SECTION 1: BACKGROUND CHARACTERISTICS

Note: The questions in Section 1 apply to all who consented to participate in this surveillance

Q No.	Questions and filters	Responses and Codes		Skip to
**Q101**	What is your age in completed years?	Age In Years [ ]		
**Q102**	Are you currently carrying a card or other document like AADHAR/PAN/WHITE CARD, where your birth date is written down?<br>IF YES: May I see the card or other document where the date of birth is written down?	Yes, Seen	1	
		Yes, Not Seen	2	
		No Card/Document	3	
☞ **If the document is seen, COMPARE AND CORRECT Q101 IF INCONSISTENT.**				
**Q103**	What has been your highest level of educational attainment?	Illiterate	1	
		Literate and till 5th Standard	2	
		6th to 10th Standard	3	
		11th to graduation	4	
		Post-Graduation and above	5	
		No Response	99	
**Q104**	What is your current primary occupation?	Agricultural Labourer	1	
		Non-Agricultural Labourer	2	
		Domestic Servant	3	
		Skilled/Semi-skilled worker	4	
		Petty business/small shop	5	
		Large Business/Self-employed	6	
		Service (Government or Private)	7	
		Student	8	→ **Q 107**
		Truck Driver/Helper	9	
		Auto/taxi driver	10	
		Hand cart pullers/rickshaw pullers	11	
		Hotel staff	12	
		Agricultural cultivator/landholder/	13	
		Unemployed	14	→ **Q 107**
		Mangati	15	→ **Q 107**
		Badhai	16	→ **Q 107**
		Others	17	
		17.a Others, Specify ____________		
		No Response	99	
**Q105**	Do you usually work throughout the year, or do you work seasonally, or only once in a while?	Throughout The Year	1	
		Seasonally/Part of The Year	2	
		Once In a While	3	
		No Response	99	
**Q106**	Are you paid in cash or kind for this work, or are you not paid at all?	Cash Only	1	
		Cash And Kind	2	
		In-Kind Only	3	
		Not Paid	4	
		No Response	99	

**Q107**	What is your current marital status?	Never Married<br>Currently Married<br>Divorced/ Separated/ Widower<br>No Response	1<br>2<br>3<br>99	

SECTION 2: PARTNER STATUS

Now, I would like to ask you some questions relating to your life/partner testing and HIV status. Let me assure you again that your answers are completely confidential and will not be disclosed to anyone. If you do not want to answer, just let me know, and I will skip to the next question.

**Q201**	Has your partner ever been tested for HIV?	Yes<br>No<br>Not Applicable<br>No Response/Don't Know	1<br>2<br>98<br>99	 **→Q301**<br>**→Q301**<br>**→Q301**
**Q202**	What was the test results?	Positive<br>Negative<br>Result not collected<br>No Response/Don't Know	1<br>2<br>3<br>99	

SECTION 3: LIFESTYLE and RELATED HEALTH ISSUES

Now I would like to ask you some questions relating to lifestyle and health matters. Let me assure you again that your answers are completely confidential and will not be told to anyone. If you do not want to answer, just let me know, and I will skip to the next question.

**Q No.**	**Questions and filters**	**Responses and Codes**		**Skip to**
**Q301**	Do you currently smoke or use tobacco in any other form?	Yes<br>No<br>No Response	1<br>2<br>99	 **→Q304**<br>**→Q304**
**Q302**	How frequently do you currently smoke or use tobacco in any other form	Every Day<br>Some Days<br>Occasionally<br>No Response	1<br>2<br>3<br>99	
**Q303**	In what other form do you currently smoke or use tobacco?<br><br>Any other form?<br><br>**RECORD ALL MENTIONED.**	Cigarettes<br>Bidis<br>Gutka / Paan Masala with<br>Tobacco<br>Khaini<br>Paan With Tobacco.<br>Other Chewing Tobacco<br>Others Specify -------------------<br>No Response	A<br>B<br>C<br>D<br>E<br>F<br>X<br>XA<br>99	
**Q304**	Do you drink alcohol?	Yes<br>No<br>No Response	1<br>2<br>99	 **→Q307**<br>**→Q307**

**Q305**	How often do you drink alcohol?	Every Day<br>Some Days<br>Occasionally<br>No Response	1<br>2<br>3<br>99	
**Q306**	What type of alcohol do you usually drink?<br><br>**RECORD ALL MENTIONED**	Tadi Madi<br>Country Liquor<br>Beer<br>Wine<br>Others<br>Specify ------------------<br>No Response	A<br>B<br>C<br>D<br>X<br>XA<br>99	
**Q307**	Are you currently injecting yourself with any drug for non-medical purposes, i.e., for pleasure?	Yes<br>No<br>No Response	1<br>2<br>99	 **→Q311**<br>**→Q311**
**Q308**	How frequently do you inject yourself with any drug for non-medical purposes, i.e., for pleasure?	Every Day<br>Some Days<br>Occasionally<br>No Response	1<br>2<br>3<br>99	
**Q309**	When was the last time you injected yourself with any drug for non-medical purposes, i.e., for pleasure?	Less than a week<br>One week to less than a month<br>More than a month ago<br>No Response	1<br>2<br>3<br>99	
**Q 310**	When you injected last for non-medical purposes, i.e., for pleasure, did you use a new needle/syringe for injecting yourself?	Yes<br>No<br>No Response	1<br>2<br>99	

Now, I would like to ask you some questions relating to common health issues. Let me assure you again that your answers are completely confidential and will not be told to anyone. If you do not want to answer, just let me know, and I will skip to the next question.

**Q No.**	**Questions, Responses and Codes**			
	Q 311: Do you currently have?		Q 312. Have you sought treatment for this problem?	
a. Diabetes?	Yes<br>No<br>Don't know/ No Response	1<br>2<br>99	Yes<br>No<br>Don't know/ No Response	1<br>2<br>99
b. Hypertension?	Yes<br>No<br>Don't know/ No Response	1<br>2<br>99	Yes<br>No<br>Don't know/ No Response	1<br>2<br>99
c. Any heart disease?	Yes<br>No<br>Don't know/ No Response	1<br>2<br>99	Yes<br>No<br>Don't know/ No Response	1<br>2<br>99
d. A chronic respiratory disease, including asthma?	Yes<br>No<br>Don't know/ No Response	1<br>2<br>99	Yes<br>No<br>Don't know/ No Response	1<br>2<br>99
e. Any chronic liver disorders?	Yes<br>No	1<br>2	Yes<br>No	1<br>2

	Don't know/ No Response	99	Don't know/ No Response	99
f. Any chronic kidney disorders?	Yes<br>No<br>Don't know/ No Response	1<br>2<br>99	Yes<br>No<br>Don't know/ No Response	1<br>2<br>99
g. Cancer?	Yes<br>No<br>Don't know/ No Response	1<br>2<br>99	Yes<br>No<br>Don't know/ No Response	1<br>2<br>99

SECTION 4: SEXUAL HEALTH AND SEXUAL HISTORY

☞ **CHECK FOR THE PRESENCE OF OTHERS. BEFORE CONTINUING, MAKE EVERY EFFORT TO ENSURE PRIVACY.** We would like to know about your sexual life. Let me assure you again that your answers are completely confidential and will not be told to anyone.

**Q401**	Have you ever had sexual intercourse?	Yes<br>No<br>No Response	1<br>2<br>99	 **→Q501**<br>**→Q501**
**Q402**	How old were you when you had sexual intercourse for the very first time?	Age In Years [ ]<br>Don't Remember / No Response	 99	
**Q403**	Have you had sex in the last 12 months?	Yes<br>No<br>Don't Remember / No Response	1<br>2<br>99	 **→Q501**<br>**→Q501**
**Q404**	When was the last time you had sex in the last 12 months?	Within a month<br>Between 1 month and 3 months<br>More than three months ago<br>Don't Remember / No Response	1<br>2<br>3<br>99	
**Q405**	Do you use condoms during sexual intercourse?	Yes<br>No<br>Don't Remember / No Response	1<br>2<br>99	 **→Q408**<br>**→Q408**
**Q406**	How frequently have you used condoms during sexual intercourse in the last three months?	Always<br>Often.<br>Sometimes<br>Don't Remember / No Response	1<br>2<br>3<br>99	
**Q407**	When you had your last sexual intercourse, did you use a condom?	Yes<br>No<br>Don't Remember / No Response	1<br>2<br>99	
**Q408**	How would you describe your relationship with the person with whom you had your last sexual intercourse?	Spouse<br>Lover<br>Casual partner<br>Paying Partner<br>Don't Remember / No Response	1<br>2<br>3<br>4<br>99	
**Q409**	Besides this person with whom you had sex last, did you have sex with any other partner in the last 12 months?	Yes<br>No<br>Don't Know / No Response	1<br>2<br>99	 **→Q501**<br>**→Q501**
**Q410**	Have any of your sexual partners paid you for sex (in cash/in-kind) in the last 12 months?	Yes<br>No<br>Don't Know / No Response	1<br>2<br>99	

SECTION 5: VIOLENCE, STIGMA AND DISCRIMINATION

People who are living with HIV/AIDS experience increased vulnerability to violence or are seen/treated in an improper manner due to the stigmatized nature of the disease and the perceived lack of social acceptance of the behaviours associated with the disease. Now, I would like you to ask some questions to understand if you have experienced any **violence, stigma, and discrimination** in the community **because of your HIV Status.** Let me assure you again that your answers are completely confidential and will not be told to anyone. If you do not want to answer, just let me know, and I will skip to the next question.

Q No.	Questions and filters	Responses and Codes		Skip to
	EXPERIENCE OF PHYSICAL VIOLENCE			
**Q501**	In the last 12 months, has anyone physically hurt you, such as hit or choked you or threatened you with a knife or other weapon because of your HIV Status?	Yes<br>No<br>I have not disclosed my HIV status to anyone<br>Don't Remember / No Response	1<br>2<br>3<br>99	 **→Q505**<br>**→Q505**<br>**→Q505**
**Q502**	In the last 12 months, how many times has anyone physically hurt you, such as hit or choked you or threatened you with a knife or other weapon?	Once<br>2–5 times<br>6–10 times<br>10 or more times<br>Don't Remember / No Response	1<br>2<br>3<br>4<br>99	
**Q503**	The last time this happened, what was your relationship with the person who did any of these things to you? If it was more than one person, what was your relationship with the person you remember best?	Sexual Partner<br>Relative<br>Friend/Acquaintance<br>Other<br>Don't Remember / No Response	1<br>2<br>3<br>4<br>99	
**Q504**	After someone did any of those things to you, did you ever try to seek professional help or services from any of the following? **CHECK ALL THAT APPLY**	I Did Not Try to Seek Help<br>Healthcare Professional<br>Social Worker, Counsellor, or NGO<br>Police Or Other Security Personnel<br>Others<br>Don't Remember / No Response	A<br>B<br>C<br>D<br>E<br>99	
	STIGMA			
**Q505**	I have lost respect or standing in the community because of my HIV status.	Strongly Agree<br>Agree<br>Neither Agree or Disagree<br>Disagree<br>Strongly Disagree<br>No Response	1<br>2<br>3<br>4<br>5<br>99	
**Q506**	I think less of myself because of my HIV status.	Strongly Agree<br>Agree<br>Neither Agree or Disagree<br>Disagree<br>Strongly Disagree<br>No Response	1<br>2<br>3<br>4<br>5<br>99	

**Q507**	I have felt ashamed because of my HIV-positive status.	Strongly Agree<br>Agree<br>Neither Agree or Disagree<br>Disagree<br>Strongly Disagree<br>No Response	1<br>2<br>3<br>4<br>5<br>99	
Sometimes HIV, infected people might have faced some unpleasant experiences in the community because of their HIV status. Now, I would ask some questions to know if you also had such experiences. Please tell us the following things have happened to you, or whether you think they have happened to you, because of your HIV status in the **last 12 months.**				
**Q508**	People have talked badly about me because of my HIV status.	Never<br>Once<br>A Few Times<br>Not Applicable Because No One Knows My HIV Status<br>No Response	1<br>2<br>3<br>4<br>99	
**Q509**	I have been verbally insulted, harassed or threatened because of my HIV status.	Never<br>Once<br>A Few Times<br>Not Applicable Because No One Knows My HIV Status<br>No Response	1<br>2<br>3<br>4<br>99	
**Q510**	I have been physically assaulted because of my HIV status.	Never<br>Once<br>A Few Times<br>Not Applicable Because No One Knows My HIV Status<br>No Response	1<br>2<br>3<br>4<br>99	
**Q511**	I have felt that people have not wanted to sit next to me, for example, on public transport, at church or in a waiting room because of my HIV status.	Never<br>Once<br>A Few Times<br>Not Applicable Because No One Knows My HIV Status<br>No Response	1<br>2<br>3<br>4<br>99	
**Q512**	Someone else disclosed my HIV status without my permission.	Never<br>Once<br>A Few times<br>Not Applicable Because No One Knows My HIV Status<br>No Response	1<br>2<br>3<br>4<br>99	

SECTION 6: HIV Testing and Treatment History<br>Many thanks for all your support in helping us understand the PLHIV community and their issues so far. Now, I would like to know something about your HIV testing and treatment history based on the documents available to you. We also measure your height, weight and blood pressure using standardized instruments and then, finally, blood samples. Results of all of the blood samples will be shared with you through your health care provider to ensure initiation of appropriate follow-up testing and treatment services if required. Let me assure you again that your answers are completely confidential and will not be told to anyone. If you do not want to answer, just let me know, and I will skip to the next question.				
**Q No.**	**Questions and filters**	**Responses and Codes**		**Skip to**
**Q 601**	Date of confirmed HIV test (DD/MM/YYYY)	☐☐ / ☐☐ / ☐☐☐☐		
**Q 602**	HIV Type	HIV 1<br>HIV 2<br>HIV 1 & 2	1<br>2<br>3	
**Q 603**	Baseline CD4 count (Number)	☐<br>CD4 Count Not Done ☐		
**Q 604**	Date of last viral load count (DD/MM/YYYY)	☐☐ / ☐☐ / ☐☐☐☐<br>Viral Load Count Not Done ☐		→ **Q606**
**Q 605**	Last tested Viral Load (Number)	☐<br>Target Not Detected (TND) ☐		
**Q 606**	Current Treatment Status	On ART<br>On Pre-ART<br>Not on ART	1<br>2<br>3	 → **Q609**<br>→ **Q609**
**Q607**	Current Drug/ Medicine in Anti-Retroviral TreatmentRegimen (Record from the white card of the respondent)	TDF+3TC+DTG (TLD)<br>AZT+3TC+DTG (ZLD)<br>ABC+3TC+DTG (ALD)<br>AZT+3TC+ATV/r(ZL+ATV/r)<br>TDF+3TC+ATV/r(TL+ATV/r)<br>TLD+DRV/RTV+DTG<br>TL+LPV/r<br>ZL+LPV/r<br>AL+LPV/r<br>Others, specify Others<br>Specify____________________	1<br>2<br>3<br>4<br><br>5<br>6<br>7<br>8<br>9<br>10	

**Q608**	Record the date of initiation of your current regimen.	☐☐ / ☐☐ / ☐☐☐☐		
**For Questions 609 – 612, the values must be measured on the day of the interview using the height scale, weighing machine and BP apparatus provided for the IBBS-PLHIV surveillance and the values must be entered in appropriate units**				
**Q609**	Measured Height (in cm)	☐☐☐ cms		
**Q610**	Measured Weight (in kg)	☐☐☐ kgs		
**Q611**	Measured Blood Pressure (Systolic) in mm	☐☐☐ mm		
**Q612**	Measured Blood Pressure (Diastolic) in mm	☐☐☐ mm		
**Q613**	How long before did you have your last meal?	☐☐ hrs ☐☐ mins		
**For Question 613, Ask the respondent, before how long they had their last meal and enquire the time. Calculate the duration between their last meal and the time of the interview. Enter the duration in hrs and mins in the space provided. Refer to the examples in the manual for further clarification.**<br><br>**Note: Do Not enter the time of their last meal for this question**				
Thank you very much for your time and for providing the information and biological samples. I assure you once again that none of the information you have given us will be shared with anyone else, and your responses will remain completely confidential				

CONFIDENTIAL: ONLY FOR THE USE OF IBBS-PLHIV SITE PERSONNEL				
**Q700**	**COMPLETION STATUS**<br>Collect the bio-behavioural data as per the protocol. Complete question 700 as per the final status of bio-behavioural data collection.			
Q700 A	Completed Interview	Yes<br>No	1<br>2	
Q700 B	Provided Blood Sample	Yes<br>No	1<br>2	
Q700 C	Provided Height and Weight	Yes<br>No	1<br>2	
Q700 D	Measured and Entered Systolic and Diastolic BP	Yes<br>No	1<br>2	

Thank the participant for their support and cooperation and reassure them about the anonymity and confidentiality of the responses. Take them to the lab technician for blood collection. Ensure that the sample number on the data form and the blood collection vial/s is the same.

**Signature:**	**Signature:**
**Name:**	**Name:**
**(Person who filled the form)**	**(Site In-charge)**
