# Supplementary material for: Integrated Behavioral and Biological Surveillance Among People Living With HIV Visiting the Antiretroviral Therapy Centers in India: Protocol for a Cross-Sectional Surveillance
Source: JMIR Res Protoc. 2025 May 21;14:e58252. doi: 10.2196/58252 (PMC12138296; doi:10.2196/58252)
Supplement: Multimedia Appendix 4 [file resprot_v14i1e58252_app4.docx]

| **Bio-Sample** | **Processed as** | **Transferred to** | **Biomarkers tested for** | **Tested at** | **Testing Protocol** |
| --- | --- | --- | --- | --- | --- |
| Blood collected in a suitable vacutainer  (10 mL) | Whole Blood (1 mL) | EDTA Tube | CD_4_ Count | ART center | Routine System at ART Centers |
|  | Plasma (from 1 mL sample) | Cryovials | Random Blood Sugar | ART center |  |
|  | Plasma (1.5 mL Plasma from 3 mL sample) | Cryovials | Viral Load | Routine Labs |  |
|  | Serum (2 mL serum from 5 mL Sample) | Serum Vials | Syphilis (RPR and Treponema pallidum hemagglutination assay) | Linked surveillance laboratory | Two test protocol |
|  |  |  | Hepatitis B and  Hepatitis C |  | One test protocol |
