# Supplementary material for: Integrated Behavioral and Biological Surveillance Among People Living With HIV Visiting the Antiretroviral Therapy Centers in India: Protocol for a Cross-Sectional Surveillance
Source: JMIR Res Protoc. 2025 May 21;14:e58252. doi: 10.2196/58252 (PMC12138296; doi:10.2196/58252)
Supplement: Multimedia Appendix 5 [file resprot_v14i1e58252_app5.pdf]

Annexure 4

Key Indicators – Data Analysis Plan

Indicators		Measurement	
Levels of unsafe sexual and injecting practices among PLHIV	Percentage of PLHIV who inject drugs	Numerator	Number of PLHIV who reported injecting drug use for non-medical purposes
		Denominator	Total Number of PLHIV recruited and responded
	Percentage of PLHIV who inject drugs reporting the unsafe injecting practices	Numerator	Number of people who inject drugs who report not using sterile injecting equipment the last time they injected drugs
		Denominator	Number of people who inject drugs who reported injecting drugs
	Percentage of PLHIV who report condomless sex act	Numerator	Number of respondents who report not using a condom the last time they had sex
		Denominator	Total number of respondents who report that they had sex in the last 12 months
	Percent of PLHIV who report condomless sex act the last time they had sex with a non-marital, non-cohabiting partner	Numerator	Number of respondents who report not using a condom the last time they had sex with a non-marital, non-cohabiting partner
		Denominator	Total number of respondents who report that they had sex with a non-marital, non-cohabiting partner in the last 12 months
	Percentage of PLHIV who report having sex with multiple partners in the last 12 months	Numerator	Percentage of PLHIV who report having sex with multiple partners in the last 12 months
		Denominator	Total number of respondents who report that they had sex in the last 12 months
Levels of lifestyle risk-behaviours	Percentage of PLHIV who smoke or use tobacco	Numerator	Number of PLHIV who reported smoking or using tobacco
		Denominator	Total Number of PLHIV recruited and responded
	Percentage of PLHIV who smoke or use tobacco	Numerator	Number of PLHIV who reported drinking alcohol
		Denominator	Total Number of PLHIV recruited and responded
	Percentage of PLHIV at health risk due to BMI	Numerator	Number of PLHIV with BMI corresponding to overweight and obese categories
		Denominator	Total Number of PLHIV with Height, weight measured and BMI calculated

Indicators		Measurement	
Prevalence of STI coinfections, namely Syphilis, Hepatitis B, and Hepatitis C among PLHIV.	Proportion of PLHIV co-infected with Syphilis	Numerator	Total number of PLHIV who tested positive for Syphilis in RPR with a titre value > 1:8
		Denominator	Total number of PLHIV recruited and tested for Syphilis.
	Proportion of PLHIV co-infected with HBV	Numerator	Total number of PLHIV who tested positive for HBV surface antigen (HBsAg)
		Denominator	Total number of PLHIV recruited and tested for HBV
	Proportion of PLHIV co-infected with HBV	Numerator	Total number of PLHIV who tested positive for HBV
		Denominator	Total number of PLHIV recruited and tested for HBV
Prevalence of NCD comorbities, namely diabetes and hypertension among PLHIV.[1]	Proportion of PLHIV with diabetes	Numerator	Total number of PLHIV with elevated levels of blood sugar (> 200 mg/dL)
		Denominator	Total number of PLHIV recruited and tested for RBS
	Proportion of PLHIV with hypertensive condition	Numerator	Total number of PLHIV with an elevated blood pressure reading (140/90 mmHg or higher)
		Denominator	Total number of PLHIV recruited and tested for BP
Advanced HIV disease[2]	Percentage of PLHIV with advanced HIV disease during the surveillance	Numerator	Number of PLHIV with CD4 < 200 cells/mm^3
		Denominator	Number of PLHIV with CD4 tested during the surveillance
Viral Load Suppression[3]	Percentage of PLHIV with viral load suppressed during the surveillance	Numerator	Number of PLHIV with VL < 1000 copies
		Denominator	Number of PLHIV with VL measured during the surveillance

[1] National AIDS Control Organization (2021). National Guidelines for HIV Care and Treatment, 2021. New Delhi: NACO, Ministry of Health and Family Welfare, Government of India.

[2] WHO Global HIV Programme.

[3] National AIDS Control Organisation (2023). Sankalak: Status of National AIDS & STD Response (Fifth edition, 2023). New Delhi: NACO, Ministry of Health and Family Welfare, Government of India.

Indicators		Measurement	
Evidence on violence, internalized stigma and discrimination	Percentage of PLHIV experiencing internalized stigma	Numerator	Number of PLHIV who agreed/strongly agreed with the one or more statement related to perceived stigma
		Denominator	Total Number of PLHIV recruited and responded
	Percentage of PLHIV experiencing stigma and discrimination in community settings	Numerator	Number of PLHIV who agreed/strongly agreed with one or more statement related to unpleasant experiences in community settings
		Denominator	Total Number of PLHIV recruited and responded
	Percentage of PLHIV experiencing physical violence	Numerator	Number PLHIV who reported experiencing physical violence in the last 12 months
		Denominator	Total Number of PLHIV recruited and responded
	Percentage of PLHIV seeking professional help or services for violence	Numerator	Number of respondents who experienced violence and sought help
		Denominator	Number PLHIV who reported experiencing physical violence in the last 12 months
Cervical cancer screening and treatment among female PLHIV	Percentage of female PLHIV screened for Cervical cancer screening	Numerator	Number of female PLHIV who were screened for cervical cancer
		Denominator	Total Number of female PLHIV recruited and responded
	Percentage of female PLHIV treated for Cervical cancer	Numerator	Number of female PLHIV screened positive for cervical precancer/cancer who received treatment
		Denominator	Total Number of female PLHIV who were screened positive for cervical cancer / precancer
